# A pilot randomized controlled trial of supervised, at-home, self-administered transcutaneous auricular vagus nerve stimulation (taVNS) to manage long COVID symptoms

**DOI:** 10.1186/s42234-022-00094-y

**Published:** 2022-08-25

**Authors:** Bashar W. Badran, Sarah M. Huffman, Morgan Dancy, Christopher W. Austelle, Marom Bikson, Steven A. Kautz, Mark S. George

**Affiliations:** 1 Neuro-X Lab, Department of Psychiatry and Behavioral Sciences, Medical University of South Carolina, Charleston, SC USA; 2 Brain Stimulation Division, Department of Psychiatry and Behavioral Sciences, Medical University of South Carolina, Charleston, SC USA; 3 Department of Biomedical Engineering, The City College of New York of CUNY, New York, NY USA; 4 Ralph H. Johnson VA Medical Center, Charleston, SC USA; 5 Department of Health Sciences and Research, Medical University of South Carolina, Charleston, SC USA

**Keywords:** COVID-19, SARTS-CoV-2, Long COVID, Post-acute sequelae of SARS-CoV-2 infection, PASC, taVNS, tVNS

## Abstract

**Background:**

Although the coronavirus disease 19 (COVID-19) pandemic has now impacted the world for over two years, the persistent secondary neuropsychiatric effects are still not fully understood. These “long COVID” symptoms, also referred to as post-acute sequelae of SARS-CoV-2 infection (PASC), can persist for months after infection without any effective treatments. Long COVID involves a complex heterogenous symptomology and can lead to disability and limit work. Long COVID symptoms may be due to sustained inflammatory responses and prolonged immune response after infection. Interestingly, vagus nerve stimulation (VNS) may have anti-inflammatory effects, however, until recently, VNS could not be self-administered, at-home, noninvasively.

**Methods:**

We created a double-blind, noninvasive transcutaneous auricular VNS (taVNS) system that can be self-administered at home with simultaneous remote monitoring of physiological biomarkers and video supervision by study staff. Subsequently, we carried out a pilot (*n* = 13) randomized, sham-controlled, trial with this system for four weeks to treat nine predefined long covid symptoms (anxiety, depression, vertigo, anosmia, ageusia, headaches, fatigue, irritability, brain fog). No in-person patient contact was needed, with informed consent, trainings, ratings, and all procedures being conducted remotely during the pandemic (2020–2021) and equipment being shipped to individuals’ homes. This trial was registered on ClinicalTrials.gov under the identifier: NCT04638673 registered November 20, 2020.

**Results:**

Four-weeks of at-home self-administered taVNS (two, one-hour sessions daily, delivered at suprathreshold intensities) was feasible and safe. Although our trial was not powered to determine efficacy as an intervention in a heterogenous population, the trends in the data suggest taVNS may have a mild to moderate effect in reducing mental fatigue symptoms in a subset of individuals.

**Conclusions:**

This innovative study demonstrates the safety and feasibility of supervised self-administered taVNS under a fully contactless protocol and suggests that future studies can safely investigate this novel form of brain stimulation at-home for a variety of neuropsychiatric and motor recovery applications.

## Background

The worldwide impact of the novel coronavirus disease (COVID-19) has been well documented over the prior two years of what became a global pandemic (Ciotti et al. 2020), (Sohrabi et al. 2020). The acute clinical symptoms of COVID-19 that occur immediately upon infection with the SARS CoV-2 virus fall on a broad spectrum – from asymptomatic to severe respiratory and cardiac distress leading to hospitalization and death (Pascarella et al. 2020), (Yuki et al. 2020). These acute symptoms may be managed by early interventions that reduce symptom severity and mortality (Struyf et al. 2021). Although progress has been made in treating acute symptoms, persistent secondary symptoms arising from COVID-19 infection, referred to as Long COVID, endure for weeks to months after infection with limited effective treatments (Silva Andrade et al. 2021). Long COVID likely involves a complex heterogenous symptomology and can lead to increased time away from work and disability (Proal 2021).

The World Health Organization defines Long COVID as “a condition that occurs in individuals with a history of probable or confirmed SARS CoV-2 infection, usually 3 months from the onset of COVID-19, with symptoms and that lasts for at least 2 months and cannot be explained by an alternative diagnosis.” Long COVID has many symptoms, often involving central and peripheral systems and results in fatigue, respiratory difficulty, musculoskeletal pain, loss of taste and smell, fever, brain fog, and new onset of neuropsychiatric conditions like anxiety and depression (Crook et al. 2021), (Cullen et al. 2020), (Moghimi et al. 2021), (Pfefferbaum and North 2020), (Troyer et al. 2020). Although the pathophysiology and reason for the persistence of these Long COVID symptoms is still unclear, symptoms that continue or develop may be due to sustained inflammatory responses and prolonged immune response after infection (Peluso et al. 2021).

Non-invasive brain stimulation (NIBS) has been suggested as a possible approach to manage COVID-19 (Adair et al. 2020), (Baptista et al. 2020), (Pilloni et al. 2020). Various NIBS techniques have demonstrated an anti-inflammatory effect, with a reduction of pain symptoms and improvement in cognition, pointing towards their potential ability to assist in the management of neuropsychiatric disorders. Of particular interest is the NIBS modality known as transcutaneous auricular vagus nerve stimulation (taVNS). taVNS involves stimulation of the auricular branch of the vagus nerve (ABVN) that bilaterally innervates the human ear (Adair et al. 2020), (Badran 2019), (Badran et al. 2018a, 2018b)–(Badran et al. 2019), (Farmer et al. 2020), (Frangos et al. 2015), (Peuker and Filler 2002). Stimulation of the ABVN has demonstrated promising anti-inflammatory (Liu et al. 2020), (Zhang et al. 2009), anti-pain (Busch et al. 2013), and antidepressant effects (Austelle 2021). These effects are driven via the vagus nerve, activating both central and peripheral mechanisms that change behavior. Relative to other neuromodulation interventions, taVNS offers in principle advantages for home-use including self-administration, battery powered device, low cost, and a robust tolerability safety profile. However, most taVNS trials have been clinic based. Transition of clinical NIBS technology from laboratory to home use requires consideration of device and protocol suitability, and taVNS protocols offer potential advantages to many NIBS technologies because they are especially linked to biomarker (e.g., heart rate, blood pressure) monitoring (Ardesch et al. 2007), (Geng et al. 2022) which facilitate safety monitoring.

The onset of the COVID-19 pandemic provided an opportunity to accelerate investigating the use of completely remote, self-administered, telemedicine approaches to brain stimulation studies. Building on that concept, we created an at-home, remote-monitored, self-administrable taVNS system, integrated with telehealth and biomarker monitoring, to manage symptoms associated with Long COVID in individuals who had prior infection with SARS CoV-2 virus. We investigated whether remote-monitored taVNS was feasible, tolerable, and whether taVNS self-administered twice daily for up to four weeks reduced symptoms associated with Long COVID.

## Methods

### Study overview

**Fig. 1:**
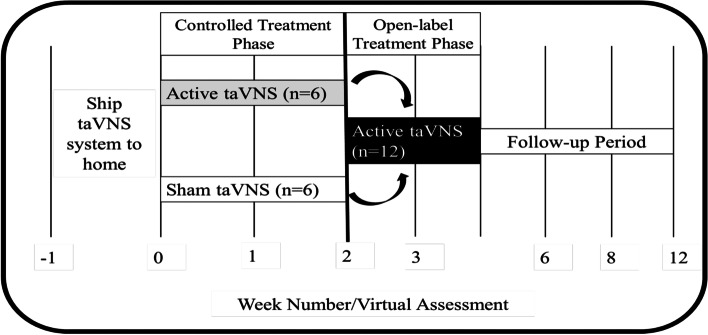
Study Timeline and Overview. Participants were shipped a taVNS kit to self-administer in their homes. After receiving the kit, they received either two weeks of either active or sham taVNS. Subsequently, all participants received two additional weeks of active taVNS stimulation

We conducted a 4-week, double-blind, sham-controlled, transcutaneous auricular vagus nerve stimulation (taVNS) trial exploring the effects of at-home, self-administered taVNS to manage long COVID symptoms (Fig. 1). This study was designed as a two-part study, part one being a randomized, double-blind, controlled trial lasting two weeks, followed by part two in which all participants received two weeks of active taVNS in an open-label design. All taVNS was self-administered by participants in their home or office space, twice daily, six days a week, for the duration of the four-week trial. This study was approved by the MUSC Institutional Review Board (IRB) and is registered on ClinicalTrials.org (NCT04638673). All participants signed written informed consent.

### Participants and inclusion criteria

We consented 13 individuals, all of whom had previously tested positive for COVID-19 (via a laboratory rapid antigen test or polymerase chain reaction (PCR) test) into this at-home taVNS trial. Because this study was conducted during the COVID-19 pandemic from November 2020 to August 2021, no procedures were conducted in person. All study procedures conducted by participants (consent, outcome measures, stimulation, and vital monitoring) were completed via virtual methods (e-mail, telephone, HIPAA-compliant video conferencing), and the taVNS devices and monitoring equipment were shipped to their home.

The inclusion criteria were as follows: over the age of 18; laboratory-confirmed COVID-19 infection; afebrile; new onset of at least one of the following nine neuropsychiatric symptoms associated with long COVID (anxiety, depression, vertigo, anosmia, ageusia, headaches, fatigue, irritability, brain fog); no damage to either left or right ear anatomy; no unstable hemodynamic effects; no ischemic or haemorrhagic stroke after developing COVID-19; ability to provide informed consent, follow instructions, read, write and speak English; attest to having reliable access to Wi-Fi internet at home.

### Mobile neurostimulation briefcase containing all study-related equipment

**Fig. 2:**
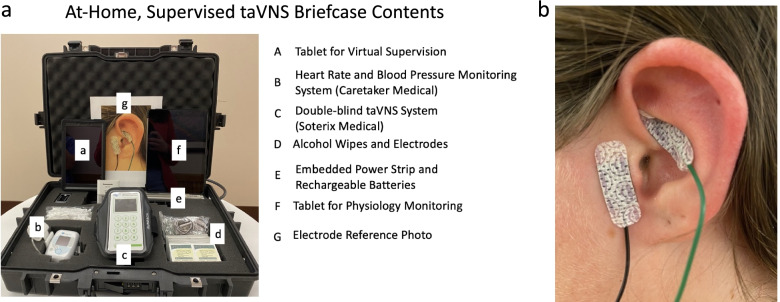
Overview of Stimulation Methodology. **A** we created an at-home taVNS kit that included all the components required to safely self-administer taVNS, as well as real-time monitor safety via physio monitoring. **B** taVNS was administered to participant’s left ear, with the anode placed on the cymba conchae of the ear, and the cathode on the tragus

To safely and effectively deliver taVNS at-home, in a manner that is user-friendly and portable, the study team designed a hard-shell briefcase containing all study related equipment. This included stimulation equipment, real time physiology monitoring equipment, and telecommunications devices required for remote monitoring (Fig. 2a).

A detailed list of the contents of the briefcase are as follow: one taVNS stimulator (Soterix Medical, Inc), 100 + pre-packaged taVNS electrodes (Neotech Products, LLC), 100 + alcohol prep pads (70% ETOH), six rechargeable lithium ion AAA batteries and charger, one vital monitoring tablet (Caretaker, LLC), vital monitoring device (Caretaker, LLC) (Gratz et al. 2017), three vital monitoring finger cuffs (Caretaker, LLC), pulse oximeter (Caretaker, LLC), one iPad for telehealth visits (Apple, Inc), embedded surge protector, one exercise arm band to facilitate portability of taVNS, one enlarged reference photo demonstrating correct taVNS electrode placement.

### Initial orientation and virtual training

After completing the virtual consent and baseline visit utilizing HIPAA-compliant telemedicine software (Doxy.me, Inc.), subjects were informed that they would be shipped a neurostimulation briefcase and simultaneously e-mailed three training videos that they were instructed to watch prior to a planned future orientation session. These videos covered overall guidance on how to use the mobile neurostimulation briefcase, including: 1) welcome and unboxing of briefcase, 2) how to use the vital monitoring device, and 3) how to use the taVNS device.

After receiving the briefcase, participants were instructed to charge all internal electronic components. Subsequently, participants then attended a virtual orientation session with a study team member during which they were trained on how to use the systems provided in the briefcase. During this orientation, which was conducted 1:1 with the participant, the study team covered three main overarching topics: 1) the purpose of each piece of equipment; 2) how to operate the vital monitoring device; 3) how to place the taVNS electrodes, turn on the stimulator and input the code to activate the stimulation device for a treatment.

Upon successful completion of this orientation visit, the study team scheduled the first treatment visit. Participants were instructed that they will be virtually monitored for compliance and safety during at least their first three days (six total taVNS sessions). Furthermore, participants were given instructions on how to contact the study team during normal business hours for any ongoing technical support.

### Self-administration procedures for taVNS

All taVNS was administered using adhesive hydrogel electrodes (Neotech Products, LLC) attached to the left ear of participants (Fig. 2B). First, participants were instructed to clean their ear using provided alcohol wipes (70% ETOH). Next, participants peeled the plastic lining off the electrodes and using either a mirror or digital screen, applied the electrodes to the two intended left ear targets (cymba conchae and tragus). To support proper placement, participants were instructed to refer to the enlarged reference photo demonstrating correct placement. This placement was informed based on prior trials and current flow modelling conducted by our group which demonstrated a highest likelihood of stimulation of the underlying auricular branch of the vagus nerve (ABVN) (Kreisberg et al. 2021). After electrodes were placed, participants connected the electrode wire to their taVNS system and were ready to self-administer stimulation (Anode- Cymba Conchae, Cathode – Tragus). The stimulation systems were double-blind, and required a code to initiate stimulation, which was sent to participants via email the morning of each taVNS treatment day. Once the participant received the code, they entered it into the system to receive their appropriate stimulation session (active or sham).

Stimulation compliance was assessed and monitored by the custom stimulation systems developed and utilized in this trial. The device monitors the real-time impedance as well as total time stimulation was administered each session, and outputs this data to the study team to provide insight to participant compliance.

### taVNS parameter settings and blinding

All taVNS sessions utilized the following parameters: 25 Hz, 500us pulse width, tonically on for 1 h, twice per day, 6 days per week. Current intensity was set to 2X individual perceptual threshold (PT), self-determined using the parametric estimation via sequential testing methods described in our prior work (Badran 2019). Perceptual threshold was conducted once, and the current intensity was pre-set for the entire study. After the PT was established, the research staff recorded which blinded group the participants were assigned to (A or B) and issued the participant group-specific stimulation codes. Neither the staff nor the participants knew that group A codes were programmed to deliver sham stimulation (0 mA of stimulation), whereas group B codes were programmed to deliver active stimulation (2X PT mA). The device screen indicated stimulation was being administered with a real-time 60-min countdown regardless of group. This code system enabled the participant and research staff to remain blinded throughout the experiment. Furthermore, codes being required to activate the device enabled compliance monitoring and regulated the number of stimulation sessions a per day.

It is important to note that participants were informed at consent that they would receive either active or sham taVNS during the initial randomized period of the trial, and it was explained that many people habituate or don’t feel stimulation after a certain period of time, often minutes. Thus, individuals expectation to perceive a noxious or persistent sensory experience was controlled between groups. The research staff interacting with the participant was not privileged to what condition the participant was receiving, as the codes were unable to be decoded to reveal condition. It is not uncommon for individuals to habituate to stimulation and not detect it on the ear, so staff was also unable to determine which group was assigned to the participant.

### Safety monitoring using a telemedicine approach

We monitored the safety of taVNS by measuring blood pressure and heart rate during the first six 1-h taVNS sessions for each participant. Safety monitoring was conducted in real-time using a dedicated tablet connected to WIFI, which communicated with a wrist-worn pneumatic device that measured heart rate and blood pressure in real time using a finger cuff attached to the middle finger of the nondominant hand of the participant (Caretaker Medical). The research staff was able to remotely connect and monitor the vitals using a web browser. Monitoring was performed for the duration of each monitored session. The study staff-maintained visual and phone communication with the participant to understand qualitative metrics of comfort, tolerability, and adverse events. All quantitative heart rate data was collected and stored for analysis of bradycardia events.

### Symptom improvement tracking and outcomes

We created a battery of outcomes to track heterogenous symptom improvements. We primarily focused on mood and anxiety effects, however due to the broad range of long-COVID impacts on the body and brain, we also included several other measures of fatigue, smell, and cognition. At inception of this study (August 2020), Long COVID was just beginning to be discussed in the literature, thus, our trial attempted capture a broad range of neuropsychiatric conditions that may have been primarily or secondarily impacted by Long COVID. This battery included the following assessments: General Anxiety Disorder-7 (GAD7), Clinical Global Impression Improvement (CGI-I), Clinical Global Impression Severity (CGI-S), Global Assessment of Functioning (GAF), COVID questionnaire, Connor Davidson Resilience Scale (CDRS), abbreviated PTSD Checklist (PCL6), Perceived Stress Scale (PSS10), Patient Health Questionnaires (PHQ4, PHQ9), pain scale, mental fatigue scale, fatigue severity scale, modified fatigue impact scale, subjective smell assessment (valence and intensity of common household items) (Konstantinidis et al. 2020), and the Montreal Cognitive Assessment (MOCA). Participants completed all assessments, which were virtually administered by research staff remotely via provided tablet. We also tracked the nine self-reported Long COVID symptoms that served as an inclusion criterion for the trial to broadly track symptom presence.

### Analytic methods

This study was designed for primary outcomes of feasibility and safety, with secondary outcomes exploring symptom improvement in nine pre-identified Long COVID symptoms. Feasibility measures were assessed by objectively rating self-administration procedures during remote-monitored pre-stimulation orientation visit, as well as the first six treatment sessions. Research staff indicated whether a participant required assistance during self-administration and scored the assistance needed for each participant for both the physiology monitoring and taVNS on a scale of 0 = no help needed, 1 = minor help needed 2 = major help needed.

Tolerability and safety outcomes were quantified by analysing participant real-time heart rate (hr) during stimulation in their first six sessions receiving at-home stimulation, regardless of randomization group assignment. For clinical measures, individual symptom improvement trends are described in a raw, individual comparison format to show trends for future trials to build upon. All statistical analysis were conducted in performed using GraphPad Prism version 8.0.0 for Windows, GraphPad Software, San Diego, California USA.

Of randomized participants, there were no dropouts, and the blind was maintained until the final participant completed all study procedures, after which the entire study team was unblinded to the conditions.

## Results

### Participants and feasibility of taVNS self-administration

We enrolled and consented 13 individuals (8 women,, however one participant was dropped out of the study before being randomized to receive any intervention due to an inability to provide positive COVID test. Thus, 12 individuals were randomized, and all participants completed the study without dropout. All 12 randomized participants (mean age 48.5 ± 11.3 years old) completed all required study visits without dropout. The mean time between the confirmed COVID + test date and enrollment date for all participants was 7.1 ± 2.75 months (min: 3 Months, max: 11 months).

**Fig. 3:**
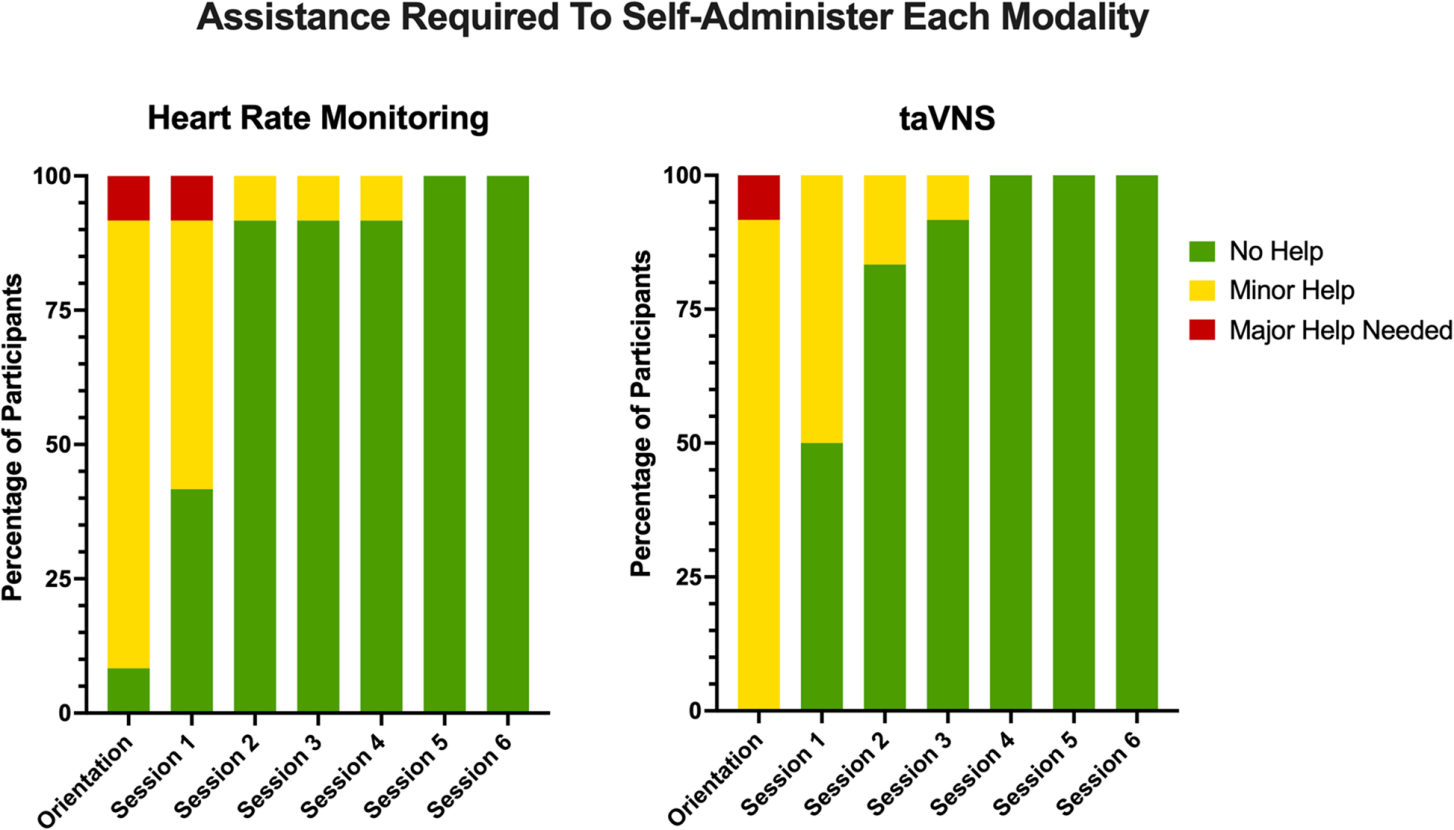
Remote Monitoring of Self-Administration Feasibility. We virtually monitored whether participants needed assistance with both the physiology monitoring and the self-administration. As demonstrated in the graphs, most participants were able to self-administer physio and taVNS proficiently within 3 sessions

Feasibility was monitored remotely, and findings reveal both the at-home heart rate monitoring and taVNS training provided before and during this trial resulted in participants who could confidently self-administer this novel intervention in a short period of time. At the initial pre-stimulation orientation period, 91% of users required assistance using the remote heart rate monitoring, and all users required assistance with taVNS setup. The amount of assistance required for both physiology monitoring and taVNS reduced each session, so that 100% of users were able to self-administer all study procedures without assistance by the 5^th^ session (third day of the 28-day intervention) (Fig. 3). No participants regressed to needing assistance in either technology.

### Compliance monitoring

Participants were asked to complete two, one-hour taVNS sessions per day, for a period of 4 weeks, with a total of 48 sessions to be administered over this period. A compliance analysis exploring the percentage of completed sessions out of the total possible number of sessions by group reveals that within the 2-week randomized period, both active and sham groups maintained a high level of compliance (sham group: 97%, active group: 97%). Compliance numbers were reduced in the subsequent 2-week open-label phase, with the initial sham group maintaining only 84% compliance, however the initially active group maintained a 94% compliance rate.

Our custom made taVNS systems monitor the real-time within-session compliance data, including time of “good” stimulation, total time of successful stimulation, and overall quality of the session. These data were analysed to determine the overall average successful minutes out of the possible 60 min taVNS session. In the double-blind phase, participants in the sham group received an average of 52.3 min of sham stimulation in each session, whereas the active group received an average of 55.8 min of active stimulation. In the open-label phase, again, use was reduced in both groups, with the original sham group receiving on average 46 min of the possible 60 min of active stimulation in each open-label session, and the original active group receiving 52.3 min of active stimulation in each open-label session.

### Tolerability and safety of at-home taVNS

Stimulation intensities were not significantly different between randomized intervention group. The mean (± SD) perceptual thresholds were Sham (*n* = 6): 0.3 ± 0.17 mA and Active (*n* = 6): 0.317 ± 0.08 mA. The treatment stimulation intensity was set to a 2X multiplier of the initial perceptual threshold value, however on rare occasions, if this dose was uncomfortable, a 0.1 mA reduction was initiated. The mean treatment intensities were Sham: 0.5 ± 0.21 mA Active: 0.6 ± 0.14 mA.

No unanticipated adverse events occurred throughout the trial. There were two instances of mild skin irritation (redness from prolonged stimulation that would not resolve for 24 h), and thus that participant switched to self-administer stimulation on the right ear until this resolved.

**Fig. 4:**
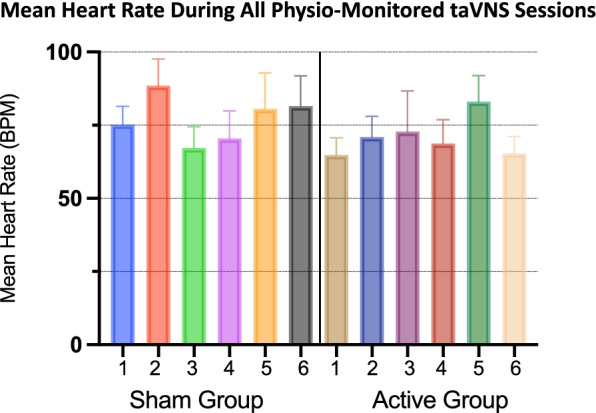
Individual Participant Heart Rate Monitoring. We recorded the mean heart rate (error bars = sem) during the first 6 taVNS sessions in all participants. Regardless of stimulation condition, all participants maintained a safe mean heart rate and did not experience any bradycardia events

taVNS did not produce any adverse cardiac events. Heart rate was recorded during the first 6 taVNS sessions (of the randomized period) throughout the duration of the treatment period. Figure 4 visualizes the average heart rate for each participant, within each condition for all heart rate measurements. All participants’ heart rates remained within the safe, normal range for adults and did not indicate any cardiac-related side effects in either sham or active group. Furthermore, we conducted an analysis of all instantaneous heart rate measurements for all participants, split into either the sham or active group (*n* = 19,244 values for sham group, *n* = 17,844 values for active group). Neither group had a bradycardic event (< 50 BPM), with the minimum HR recorded in the sham group being 67.50 BPM, and the Active group 55.33 BPM.

### Long COVID symptom improvements and individual trends in mental fatigue

**Fig. 5:**
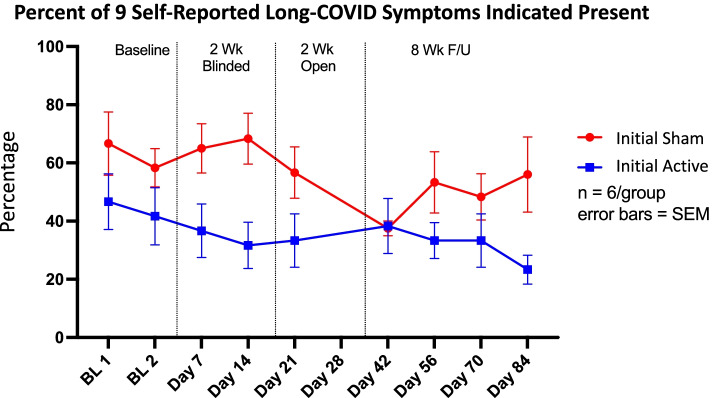
Long-COVID Symptom Improvement by Randomization Group. In this small sample, taVNS reduced the mean percent of Long-COVID symptoms experienced by participants. During the two-week blinded period, sham taVNS provided no benefit to participants, however during the open label period, those individuals reduced their Long-COVID symptom burden. In the initial active group, we see a marked reduction in Long-COVID symptoms over the course of treatment and follow-up

This trial included a large battery of clinically validated scales to measure in-depth symptomology changes, including depression, anxiety, stress, cognition, and smell. Individual analysis of these battery subcomponents revealed no significant changes in these measurements. Thus, we wholistically monitored the symptoms of nine specific Long COVID symptoms (anxiety, depression, vertigo, anosmia, ageusia, headaches, fatigue, irritability, brain fog). Due to the heterogeneity of Long COVID, participants did not present with all nine symptoms, however we calculated the overall percentage of these nine symptoms as a meta-indicator of Long COVID severity. At baseline, the sham group reported a higher number of symptoms present (sham – 66%, active 46%). At completion of the 2-week blinded phase, the sham group showed no improvement in symptoms present (68%), however the active group had a reduced number of reported symptoms (31%). After the 4-week treatment phase, both groups (initial active, initial sham) reported 38% of the nine symptoms. Interestingly, during the follow-up phase, the initial sham group who only received 2 weeks of active treatment worsened. They increased to a self-reported 56% of symptoms, whereas the initial active group who received 4 total weeks of treatment reported 23% (Fig. 5).

Although this study was not powered to demonstrate effectiveness of taVNS on any one individual symptom of Long COVID, interesting individual trends were revealed in in mental fatigue. These findings demonstrates that participants receiving an initial 2-week period of sham taVNS report moderate improvement in their mental fatigue symptoms, however those receiving the full 4-week course of active taVNS intervention demonstrated the largest reductions in mental fatigue scores.

## Discussion

In this trial, we designed and developed a completely remote, double-blind, telemedicine controlled, transcutaneous auricular vagus nerve stimulation system (taVNS) with companion real-time heart rate monitoring. We then investigated the use of this novel, self-administrable taVNS system during the early COVID-19 pandemic (2020–2021) to understand its safety, feasibility, and potential to manage Long COVID symptoms in the home setting. Each participant self-administered taVNS twice daily, in one-hour sessions, six days a week for up to 4 weeks. This completely remote and virtual (no in-person visits) brain stimulation approach was feasible, with participants learning how to reliably self-administer stimulation within their first five sessions. Furthermore, stimulation was safe, even at home, with no bradycardia or serious adverse events occurring during the stimulation sessions. Lastly, there was a mild improvement in overall self-reported Long COVID symptoms, as well as mental fatigue, although this small study was not powered to detect clinical effects.

It has been over two years since the beginning of the COVID-19 pandemic, but the persistent, long-term impacts of the SARS-CoV-2 virus are still not understood. Long COVID is a complex and heterogenous disorder that involves several central and peripheral symptoms that are difficult to individually address from a mechanistic perspective. Even with these challenges, a main underlying hypothesis is that these prolonged symptoms are due to extended neuroinflammatory response. Interestingly, although vagus nerve stimulation (VNS) has been used in humans since 1997 for treating refractory epilepsy (George et al. 2000), there recently has been increased enthusiasm surrounding the use of VNS for several inflammatory disorders in humans such as rheumatoid arthritis (Drewes et al. 2021), (Koopman et al. 2017), (Kwan et al. 2016). These anti-inflammatory effects have been validated in animal models demonstrating the cellular, molecular, and central anatomical mechanisms that mediate central modulation of immune functions suggesting attenuation of cytokine storm (Guo 2017) and activation of vagal mediated inflammatory response.

Early evidence from an alternative noninvasive VNS trial (gamma Core device targeting the cervical bundle of the vagus nerve by applying electrical stimulation to the neck landmarked by the carotid sheath) suggests that stimulation of the vagus nerve has mild anti-inflammatory effects in 97 COVID-19 patients (Tornero et al. 2022). Although non-invasive vagus nerve stimulation may be administered with a variety of different technologies, evidence suggests it potentially engages an anti-inflammatory mechanism that should be further explored in future trials.

### Limitations

This small pilot trial has several limitations, many arising from the exploratory science conducted during an unprecedented time where innovation and deployment of interventions were quite rapid. Long COVID at the time this study began, and still today, is not well understood, and the heterogeneity of the sample reduced our ability to detect any significant behavioural impact. In larger, follow-up studies, a narrower inclusion criterion may be beneficial, however this heterogeneity may introduce confounding variables in an otherwise narrow inclusion criterion. Aside from our small sample size and lack of power to determine behavioural effects, this study was intended create a new completely remote brain stimulation technology that could continue to be researched in Long COVID.

taVNS is still in its infancy as a neurostimulation modality and the dosing considerations are yet to be established. We decided to deliver two, one-hour sessions daily for four weeks. Although tolerable and safe, and demonstrated to have high compliance, this may have been burdensome to participants. Increasing the number of sessions or length of sessions may perhaps bolster behavioural effects, however skin irritation may emerge as a limiting factor.

## Conclusions

This study successfully built and deployed a completely remote, at-home, supervised taVNS system that was tolerable, safe, and used with exceptionally high compliance rates in a small sample of Long COVID patients. Over four weeks, 12 participants were able to successfully self-administer taVNS with limited training and were self-sufficient within two days. Furthermore, compliance was high, and no reports of adverse cardiac effects. Future investigations and randomized trials should be conducted using similar methodology in a larger sample of Long COVID patients.

## Data Availability

The datasets generated and/or analysed during the current study are not publicly available due to patient confidentially but are available from the corresponding author on reasonable request.

## Abbreviations

ABVN: Auricular branch of the vagus nerve
CDRS: Connor Davidson Resilience Scale
CGI-I: Clinical Global Impression Improvement
CGI-S: Clinical Global Impression Severity
COVID-19: Coronavirus disease 19
GAD7: General Anxiety Disorder-7
GAF: Global Assessment of Functioning
IRB: Institutional Review Board
MOCA: Montreal Cognitive Assessment
MUSC: Medical University of South Carolina
NIBS: Non-invasive brain stimulation
PASC: Post-acute sequelae of SARS-CoV-2 infection
PCL6: Abbreviated PTSD Checklist
PHQ4, PHQ9: Patient Health Questionnaires
PSS10: Perceived Stress Scale
PT: Perceptual threshold
TaVNS: Transcutaneous auricular VNS
VNS: Vagus nerve stimulation
